# The Effects of Curcumin on Diabetes Mellitus: A Systematic Review

**DOI:** 10.3389/fendo.2021.669448

**Published:** 2021-05-03

**Authors:** Ledyane Taynara Marton, Laís Maria Pescinini-e-Salzedas, Maria Eduarda Côrtes Camargo, Sandra M. Barbalho, Jesselina F. dos Santos Haber, Renata Vargas Sinatora, Claudia Rucco Penteado Detregiachi, Raul J. S. Girio, Daniela Vieira Buchaim, Patricia Cincotto dos Santos Bueno

**Affiliations:** ^1^ Department of Biochemistry and Pharmacology, School of Medicine, University of Marília (UNIMAR), Marília, Brazil; ^2^ Postgraduate Program in Structural and Functional Interactions in Rehabilitation-UNIMAR, Marília, Brazil; ^3^ Department of Biochemistry, School of Food and Technology of Marilia (FATEC), Marília, Brazil

**Keywords:** *Curcuma longa*, curcumin, curcuminoids, diabetes, type 2 diabetes mellitus

## Abstract

Diabetes mellitus (DM) is an ensemble of metabolic conditions that have reached pandemic proportions worldwide. Pathology’s multifactorial nature makes patient management, including lifelong drug therapy and lifestyle modification, extremely challenging. Currently, there is growing evidence about the effectiveness of using herbal supplements in preventing and controlling DM. Curcumin is a bioactive component found *Curcuma longa*, which exhibits several physiological and pharmacological properties such as antioxidant, anti-inflammatory, anticancer, neuroprotective, and anti-diabetic activities. For these reasons, our objective is to systematically review the effects of *Curcuma longa* or curcumin on DM. Databases such as PUBMED and EMBASE were searched, and the final selection included sixteen studies that fulfilled the inclusion criteria. The results showed that curcumin’s anti-diabetic activity might be due to its capacity to suppress oxidative stress and inflammatory process. Also, it significantly reduces fasting blood glucose, glycated hemoglobin, and body mass index. Nanocurcumin is also associated with a significant reduction in triglycerides, VLDL-c, total cholesterol, LDL-c, HDL-c, serum C reactive protein, and plasma malonaldehyde. Therefore, it can be considered in the therapeutic approach of patients with DM.

## Introduction

The International Diabetes Federation has estimated that since 2000, the prevalence of diabetes mellitus (DM), including type 1 (T1DM) and type 2 (T2DM), has increased from 151 million to 463 million in adults aged 20 to 79 years (1). T2DM is the most prevalent chronic metabolic condition characterized by higher blood glucose levels due to the organism’s poor management of insulin. The state of chronic hyperglycemia leads to increased levels of advanced glycation end products (AGEs) that act directly on cells, causing pro-inflammatory effects and oxidative stress (2, 3).

The triggering ways of inflammation in T2DM are still not fully elucidated. The inflammatory process likely contributes to the development of T2DM and may cause insulin resistance which is worsened with hyperglycemia (4). Epidemiologic studies have shown an association between inflammatory biomarkers and the occurrence of T2DM and its complications. Adipose tissue seems to be a significant site of inflammatory biomarkers’ production due to the cross-talk observed between adipocytes, macrophages, and other immune cells that permeate the increased adipose tissue (5, 6).

On the other hand, oxidative stress has a key role in the development of T2DM. Increased oxidative species production and reduced antioxidant capacity have been repeatedly shown in subjects with T2DM (7). Hyperglycemia can contribute to oxidative stress by enhancing the polyol pathway flux, activation of protein kinase C, altering eicosanoid metabolism, and induction of glucose autoxidation that collectively results in increased reactive oxygen species (ROS) generation. ROS can exert numerous detrimental effects that induce and aggravate diabetes, including diminishing glucose transport channels, reducing insulin secretion, protein fragmentation and oxidation, DNA damage, free fatty acid generation, and increased vascular permeability. Moreover, oxidative stress induces the formation of AGEs contributing to endothelial dysfunction and the development of microvascular and macrovascular complications of T2DM (8–10).

Owing to the aforementioned detrimental effects of oxidative stress on the development of T2DM and the progression of its vascular complications, antioxidant therapy has been considered a potentially effective approach (11, 12).

*Curcuma longa*, the turmeric plant commonly utilized in food preparation as a spice, has been recognized by the scientific community. This plant is characterized by orange tuberous rhizomes and is widely known and cultivated in South East Asia (13). It is used as a natural therapeutic medicine for various pathological conditions in these regions since ancient times. The singular characteristic of this plant is the presence of curcumin, which shows antioxidant and anti-inflammatory properties (14). Besides that, curcumin has a potential role in preventing and treating several diseases due to various actions such as anti-bacterial, anti-diabetic, anti-viral, and anticancer activities (15–17).

Curcuminoids have been shown to improve insulin resistance, decrease glucose and insulin levels, increase adiponectin release, and reduce the levels of leptin, resistin, interleukin (IL)-6 IL-1β, and tumor necrosis factor-α in patients with T2DM (18). These findings suggest that these compounds can affect glucose homeostasis and diabetic complications, and the vascular risk of patients with T2DM (19). Some studies have shown that supplementation of curcuminoids improves the lipid profile and increases the total antioxidant capacity of patients with T2DM (20–22), thus supporting other available evidence on the role of curcuminoids in modifying cardiometabolic risks (23–26). Given the above, this systematic review aimed to investigate the effects of *Curcuma longa* and its derivatives on DM.

## Methods

### Focused Question

This review was built to answer the focused question: What are the effects of *Curcuma longa* on T2DM patients?

### Language

Only studies in English were selected.

### Databases

This study has included studies in MEDLINE–PubMed (National Library of Medicine, National Institutes of Health), EMBASE, and COCHRANE databases. The descriptors used were Hyperglycemia or Diabetes or Insulin resistance and *Curcuma Longa* or Curcumin or Curcuminoids. The use of these mesh-terms helped identify studies related to curcumin intake and its beneficial role in metabolic status in T2DM patients. The authors have followed PRISMA (Preferred Reporting Items for a Systematic Review and Meta-Analysis) guidelines (27).

### Study Selection

This review included studies that reported *Curcuma longa* or potential curcumin role to patients with T2DM. This study’s inclusion criteria were Randomized Clinical Trials (RCTs), prospective, double-blind, and placebo-controlled studies. Only full texts were included.

The exclusion criteria were studies with animals, reviews, studies not in English, retrospective studies, case reports, poster presentations, and editorials. Reviews were consulted to build the discussion but were not included.

### Data Extraction

This search’s search period included the past five years (January 2016 to December 2020). These studies are described in **Table 1**.

**Table 1:** Descriptive table of the included studies.

Reference	Local	Model and Patients	Intervention	Outcomes	Adverse effects
Jiménez-Osorio et al. (28)	Mexico	Randomized double-blind placebo-controlled clinical trial with 101 individuals, 50 with non-diabetic proteinuric CKD and 51 patients with diabetic proteinuric CKD, 61 ♂ and 40 ♀ (20–70 y).	Subjects received either turmeric capsules with curcumin 107 mg in each meal (320 mg/day) or a PL for 8 w.	The intervention with curcumin did not improve proteinuria, estimated glomerular filtration rate, or lipid profile. However, curcumin attenuated lipid peroxidation in individuals with non-diabetic proteinuric CKD.	NR by the authors
Rahimi et al. (29)	Iran	Randomized double-blind placebo-control clinical trial with 80 patients with T2DM (FBG ≥ 126 mg/dl or 2-h postprandial blood glucose ≥200 mg/dl), ≥18, 31 ♂ and 39 ♀.	Subjects were assigned to nano-curcumin (as nano-micelle 80 mg/day) or PL for 3 m.	A significant decrease was observed for HbA1C, FBG, TG, and BMI after the treatment. Significant differences in HbA1c, eAG, LDL-c, and BMI variables were observed between the treated group and PL	NR by the authors
Panahi et al. (20)	Iran	Randomized double-blind placebo-controlled trial with 118 patients with T2DM (FPG ≥126 mg/dl, HbA1C ≥6.5%), or the use of standard anti-diabetic treatments, 51 ♂ and 49 ♀ 18–65 y.	Patients were randomized to curcuminoids (1000 mg/d + piperine 10 mg/d) or matching PL for a period of 12 w.	Curcuminoids lead to a significant elevation in serum total antioxidant capacity and SOD activities, while serum MDA levels were significantly reduced compared with the PL.	No severe adverse events
Panahi et al. (21)	Iran	Randomized double-blind placebo-controlled trial with 118 patients with T2DM (FPG ≥126 mg/dl, HbA1C ≥6.5%), or the use of standard anti-diabetic treatments, 51 ♂ and 49 ♀, 18–65 y.	Patients received curcuminoids (1000 mg/day + piperine 10 mg/day) or placebo/12 w.	Significant reductions in TC, non-HDL-c, and Lp(a); and elevations in serum HDL-c levels, were observed in the treated group. Serum TG and LDL-c did not show significant difference.	No severe adverse events.
Panahi et al. (30)	Iran	Randomized double-blind placebo-controlled trial with 118 patients with T2DM based (FPG ≥126 mg/dl, HbA1C ≥6.5%) or standard anti-diabetic treatments, 51 ♂ and 49 ♀, 18–65 y.	Patients were allocated to standard-of-care treatment and dietary advice plus either curcuminoids (500 mg/day + piperine 5 mg/d) or placebo for 3 m.	Significant reduction in glycemia and HbA1c were observed after curcuminoids supplementation. Additionally, participants showed lower serum AST and ALT in the treated group.	NR by patients
Adab et al. (31)	Iran	Randomized double-blind clinical trial included 80 hyperlipidemic T2DM patients (39 ♀ and 36 ♂); 30–70 y.	The patients received 2,100 mg of turmeric powder (capsules after main meals)/d/8 w.	The intervention caused a significant weight reduction, BMI, TG, and LDL-c in the intervention group compared with baseline. The intervention with turmeric powder also prevented the increase of TC.	NR by patients.
Adibian M. et al. (32)	Iran	Randomized double-blind placebo-controlled trial included 44 diabetic individuals (22 ♂, 22 ♀;40–70 y; BMI 18.5–30 kg/m^2^, with a duration of T2DM of 1 to 10 years and intake of oral hypoglycemic agents for control.	Participants were divided into curcumin group (n=21; 13 ♂ and 8 ♀) and PL group (n=23; 9 ♂ and 14 ♀). The curcumin group received 1,500 mg curcumin (500 mg capsules 3×/day)/10 w.	The intervention showed a significant decrease in TG, hs-CRP, mean FBG, and a significant increase in serum adiponectin. The curcumin group also had a significant reduction in mean weight compared with the control group.	NR by authors.
Asadi et al. (33)	Iran	Randomized double-blind parallel placebo-controlled clinical trial with 80 patients with NIDDM and DSPN (70 ♀ and 10 ♂), 30–60 y, BMI 25 to 39.9 kg/m^2^.	The participants received curcumin (Nano curcumin capsules, 80 mg/d) or PL/8 w.	After 8 w of curcumin intake, subjects showed a significant decrease in WC, FBS, HbA1c, neuropathy score, and total reflex score.	Two cases of stomachache in the first few days of the study.
Hodaei et al. (34)	Iran	Randomized double-blind placebo-controlled trial that included 53 patients with NIDDM (22 ♂ and 22 ♀; 40–70 y; BMI between 18.5 to 35 kg/m^2^.	The patients were allocated to curcumin (n=21) or PL (n=23). The intervention group received 500 mg of curcumin after each main meal/10 weeks.	The intervention showed a significant decrease in body weight, BMI, hip circumference, and FBG.	The participants did not report serious side effects.
Srinivasan et al. (35)	South India	Randomized placebo-controlled clinical trial with 136 T2DM patients (29 ♂ and 85 ♀), 30–65 y, diagnosed within the past 3 months to 10 years, on treatment with metformin for a period of at least past 3 months.	The patients received 400 mg of *Curcuma longa* 3× d (n = 60) or similar PL capsules (n = 54) for 3 m.	The *Curcuma longa* group showed a significant reduction in carotid-femoral PWV, SBP, DBP, pulse pressure, right and left brachial-ankle PWV, systolic aortic pressure, aortic pulse pressure, diastolic aortic pressure, aortic augmentation pressure, aortic augmentation index, and aortic augmentation index at heart rate 75.	One patient reported increased frequency, and one referred to upper abdominal pain.
Vanaie et al. (36)	Iran	Randomized double-blind placebo-controlled clinical trial with 60 patients with T2DM on oral anti-diabetic drugs or insulin. Curcumin group consisted of 19 patients ♀ and 27 ♂ (52–71 y).	Patients received 500 mg of curcumin with each meal (3× d after meal) or PL for 16 w.	The intervention with curcumin showed ameliorating macroscopic proteinuria in T2DM patients.	Epigastric pain in one participant.
Asadi et al. (37)	Iran	Randomized double-blind parallel and a placebo-controlled clinical trial with 80 T2DM patients with DPN, 40 in each group, 35 ♀ and 5 ♂ received nanocurcumin, and 35 ♀and 5 ♂ received PL (30–60 y).	The participants were allocated randomly to receive either 80 mg of nano-curcumin or PL capsules daily for 8 w.	It was seen a significant reduction in the mean score of depression and anxiety in the treated group compared with PL.	Two patients reported stomachache.
Funamoto et al. (38)	Japan	Randomized double-blind placebo-controlled clinical trial with 52 patients with impaired glucose tolerance or NIDDM, 23 ♂ and 10 ♀ (20–85 y).	Subjects received Theracurmin^®^ capsule (90 mg 2× d) for 6 m or PL.	Curcumin inhibited the increase in oxidized LDL-c.	NR by the authors
Shafabakhsh et al. (39)	Iran	Randomized double-blind placebo-controlled clinical trial with 60 participants with diabetes on HD (18- 80 y), 32 ♂ and 21 ♀.with 60 subjects.	Subjects were randomly separated into two groups that received 80 mg/day nano-curcumin capsule or PL for 12 w.	Nano-curcumin showed benefits on the metabolic profile in patients with diabetes on HD since it showed a significant decrease in FBG and serum insulin levels compared with PL. Nano-curcumin was also related to a significant decrease in TG, VLDL-c, TC, LDL-c, HDL-c, serum hs-CRP, and plasma MDA.	NR by patients
Shafabakhsh et al. (40)	Iran	Randomized double-blind placebo-controlled trial with 60 patients with T2DM and CHD with 2- and 3-vessel CHD, 30 individuals in each group (45–85 y).	Patients received 1000 mg/day curcumin or PL for 12 w.	Curcumin intake showed beneficial effects on PSQI, TAC, GSH, MDA values, and gene expression of PPAR-γ, but did not affect BDI, BAI, and mRNA expression for IL-1, IL-8, TGB-β, and VEGF.	NR by patients
Mokhtari et al. (41)	Iran	Randomized double-blind placebo-controlled clinical trial with 60 patients with grade 3 DFU; 39 ♂ and 11 ♀ (45–85 y).	Subjects were randomized to receive 80 mg nano-curcumin tablets daily for 12 w or PL.	Nanocurcumin intake resulted in a significant improvement of glycemic control, total- and LDL-cholesterol, TAC, and GSH.	NR by patients

BAI, Beck Anxiety Inventory; BDI, Beck Depression Inventory; BMI, body mass index; DFU, diabetic foot ulcer; CHD, coronary heart disease; CKD, chronic kidney disease; DBP, diastolic blood pressure; DPN, diabetic peripheral neuropathy; DSPN, diabetic sensorimotor polyneuropathy; eAG, estimated average glucose; FBG, fasting blood glucose; FBS, fasting blood sugar; FPG, fasting plasma glucose; GSH, glutathione; HbA1C, glycated hemoglobin; HD, hemodialysis; HDL-C, high density lipoprotein-cholesterol; hs-CRP, high-sensitivity C-reactive protein; IL-1, interleukin-1; IL-8, interleukin-8; LDL-C, low density lipoprotein-cholesterol; Lp(a), lipoprotein A; MDA, malondialdehyde; mRNA, messenger RNA; NIDDM, non-insulin-dependent diabetes mellitus; non-HDL-C, non high-density lipoprotein cholesterol; NR, not reported; PL, placebo; PPAR-g, peroxisome proliferator-activated receptor gamma; PSQI, Pittsburgh Sleep Quality Index; PWV, pulse wave velocity; RT-PCR, reverse transcription polymerase chain reaction; SBP, systolic blood pressure; SOD, superoxide dismutase; TAC, total antioxidant capacity; TC, total cholesterol; TG, triglyceride; TGF-b, transforming growth factor beta; T2DM, type 2 diabetes mellitus; T2D, type 2 diabetes; VEGF, vascular endothelial growth factor; VLDL-c, very low density lipoprotein-cholesterol; WC, waist circumstance.

### Quality Assessment

To evaluate the risk of biases in the selection, detection, and reporting bias of each.

RCT, we applied the Cochrane Handbook for Systematic Reviews of Interventions. Other risks of biases in the selection of patients, classification of interventions, missing data, and measurement of outcomes were also evaluated.

## Results

**Figure 1:**
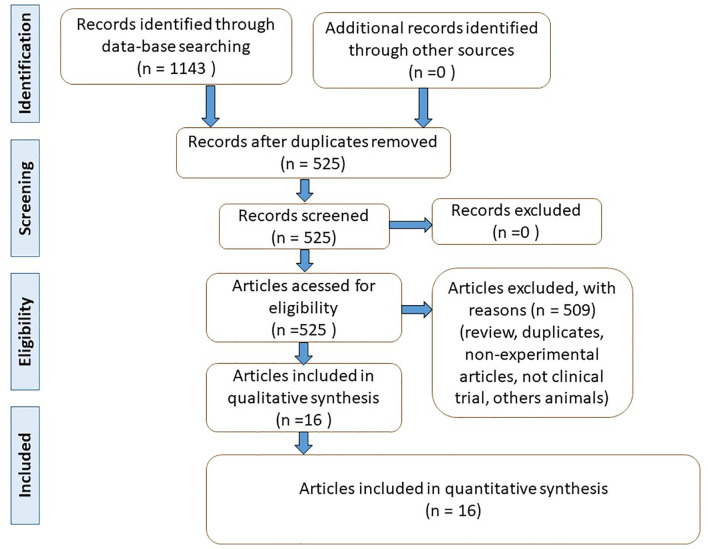
Flow diagram showing the literature search (based on PRISMA, 2009) (27).

From the 16 articles selected (**Figure 1**), 12 were randomized, double-blind placebo-controlled clinical trials, two randomized, double-blind, parallel placebo-controlled clinical trials, one randomized, double-blind clinical trial, and one randomized placebo-controlled clinical trial. A total of 1,309 participants were included, and 1,207 of those were T2DM patients. Five hundred ninety-five were women, and 495 were men. One study did not report the gender of the participants, and 12 studies reported only the gender of the participants who completed the study. The age range was from 18 to 85 years. The studies were performed in different countries (thirteen were from Iran, one from Mexico, one from South Africa, and one from Japan).

Nine of them were performed with T2DM patients, one with T2DM and non-diabetic proteinuric Chronic kidney disease (CKD) patients, one with T2DM and Diabetic Sensorimotor Polyneuropathy (DSPN) patients, one with T2DM and Diabetic Peripheral Neuropathy (DPN) patients, one with Non-Insulin-Dependent Diabetes Mellitus (NIDDM) patients, one with DM on Hemodialysis patients, one with T2DM and coronary heart disease, and one diabetic foot ulcer (DFU). Only one study included patients with type 1 and T2DM.

Five studies used curcumin, five used nano-curcumin, one used Theracurmin^®^, one *Curcuma-longa*, one turmeric powder, three curcuminoids associated with piperine. The doses administered had a wide variation range from 80 mg per day to 2,100 mg per day, and the period of intervention ranged from 8 weeks to 16 weeks

The studies showed that the use of *Curcuma longa* or curcumin (in different formulations) showed significant reduction of lipid peroxidation, fasting blood glucose levels, Glycated hemoglobin (HbA1C), triglycerides, total cholesterol, LDL-c, C-Reactive Protein, systolic and diastolic blood pressure. A significant increase in HDL-c levels and serum antioxidant capacity was also observed. The studies also showed that the use of curcumin could improve depression and anxiety levels. The primary reported adverse effects were epigastric pain and upper abdominal pain (**Table 1**). **Table 2** shows the risk of bias for the included studies.

**Table 2:** Descriptive table of the biases of the included randomized clinical trials.

Study	Question focus	Appropriate randomization	Allocation blinding	Double-blind	Losses (<20%)	Prognostics or demographic characteristics	Outcomes	Intention to treat analysis	Sample calculation	Adequate follow-up
Jiménez-Osorio et al. (28)	**Yes**	**Yes**	**Yes**	**Yes**	**?**	**Yes**	**Yes**	**No**	**NR**	**No**
Rahimi et al. (29)	**Yes**	**Yes**	**Yes**	**Yes**	**Yes**	**Yes**	**Yes**	**Yes**	**Yes**	**Yes**
Panahi et al. (20)	**Yes**	**Yes**	**Yes**	**Yes**	**Yes**	**Yes**	**Yes**	**No**	**NR**	**Yes**
Panahi et al. (21)	**Yes**	**Yes**	**Yes**	**Yes**	**Yes**	**Yes**	**Yes**	**Yes**	**NR**	**Yes**
Panahi et al. (30)	**Yes**	**Yes**	**Yes**	**Yes**	**Yes**	**Yes**	**Yes**	**Yes**	**Yes**	**Yes**
Adab et al. (31)	**Yes**	**Yes**	**Yes**	**Yes**	**Yes**	**Yes**	**Yes**	**Yes**	**Yes**	**Yes**
Adibian et al. (32)	**Yes**	**Yes**	**Yes**	**Yes**	**Yes**	**Yes**	**Yes**	**Yes**	**Yes**	**Yes**
Asadi S et al. (33)	**Yes**	**Yes**	**Yes**	**Yes**	**Yes**	**No**	**Yes**	**No**	**Yes**	**No**
Hodaei et al. (34)	**Yes**	**Yes**	**Yes**	**Yes**	**Yes**	**Yes**	**Yes**	**Yes**	**NR**	**Yes**
Srinivasan et al. (35)	**Yes**	**Yes**	**Yes**	**Yes**	**Yes**	**Yes**	**Yes**	**Yes**	**Yes**	**?**
Vanaie et al. (36)	**Yes**	**Yes**	**Yes**	**Yes**	**No**	**Yes**	**Yes**	**No**	**Yes**	**Yes**
Asadi et al. (37)	**Yes**	**Yes**	**Yes**	**Yes**	**No**	**Yes**	**Yes**	**Yes**	**Yes**	**Yes**
Funamoto et al. (38)	**Yes**	**Yes**	**Yes**	**Yes**	**No**	**Yes**	**Yes**	**No**	**Yes**	**Yes**
Shafabakhsh et al. (39)	**Yes**	**Yes**	**Yes**	**Yes**	**Yes**	**Yes**	**Yes**	**No**	**Yes**	**Yes**
Shafabakhsh et al. (40)	**Yes**	**Yes**	**Yes**	**Yes**	**Yes**	**Yes**	**Yes**	**No**	**Yes**	**Yes**
Mokhtari et al. (41)	**Yes**	**Yes**	**Yes**	**Yes**	**Yes**	**Yes**	**Yes**	**No**	**Yes**	**Yes**

NR, not reported.

## Discussion

### Type II Diabetes Mellitus in a Nutshell

T2DM is a prevalent chronic disorder characterized by high blood sugar levels representing 80% of DM patients. This condition has a multifactorial nature, triggered by several genetic and environmental factors, with a critical mechanism of progressive loss of β-cell insulin secretion (42–44). As long as the patient β-cells can compensate for insulin resistance, the normoglycemia is preserved. However, the natural disease progression is hyperglycemia (43, 45).

The chronic hyperglycemic state contributes to oxidative stress by increasing levels of AGEs and ROS (46), increasing the risk of development of microvascular (retinopathy, nephropathy, and neuropathy), and macrovascular complications (congestive heart failure, stroke, coronary heart disease, myocardial infarction, and peripheral vascular disease) (42, 47). These consequences result in a reduction in quality of life and life expectancy by even ten years among diabetic patients (42, 48).

Arterial hypertension, dyslipidemia, older age, sedentary lifestyle, and abdominal obesity are key risk factors for T2DM (43, 48) since the adipose tissue secretes several biomarkers, such as resistin, TNF-α, and IL-6, which can induce a chronic inflammatory state and insulin resistance. Moreover, in obese patients, low adiponectin levels and a leptin-resistance state are commonly seen (42).

**Figure 2:**
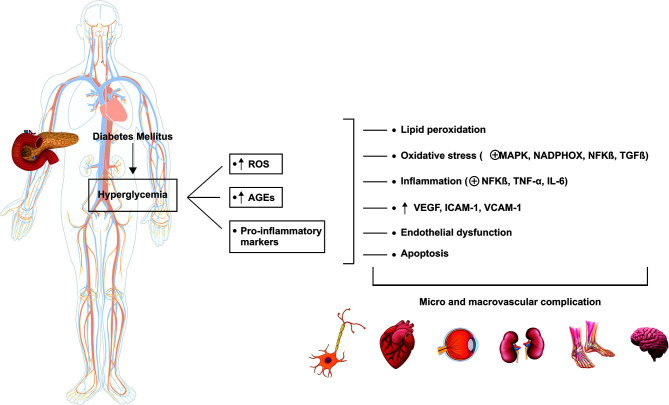
Pathophysiology of diabetes mellitus. The hyperglycemic state leads to the increase in ROS and pro-inflammatory biomarkers related to the complications associated with diabetes. ROS, reactive oxygen species; AGEs, advanced glycation end-products; MAPK, mitogen-activated protein kinase; NADPHOX, nicotinamide adenine dinucleotide phosphate oxidase; NFKβ, nuclear factor-kappa β; TGF-β, transforming growth factor β; TNF-α, tumor necrosis factor α; IL-6, interleukin-6; VEGF, vascular endothelial growth factor; ICAM-1, intercellular adhesion molecule 1; VCAM-1, vascular cell adhesion protein.

The natural progression of DM is hyperglycemia, which contributes to oxidative stress, and pro-inflammatory markers, which lead to lipid peroxidation, increase the oxidative stress scenario, resulting in inflammation and increased VEGF, ICAM-1, VCAM-1, endothelial dysfunction, and apoptosis. These processes increase the risk of micro and macrovascular complications (49–51). **Figure 2** summarizes the pathophysiology of DM.

The current DM treatment strategies are based on a multifactorial approach, targeting all risk factors rather than glucose control alone, resulting in a decrease or delay in its progression and improving the overall life quality (42, 48). However, there is no cure for diabetes so far, and none of the large numbers of the drug classes that are used modifies the progressive decline in β-cell function over time (42, 43). Therefore, researchers have been searching for evidence about herbal therapy’s effectiveness in preventing and controlling DM.

### Curcuma longa

The genus *Curcuma* (Zingiberaceae) includes perennial rhizomatous plants native to subtropical to tropical regions. *Curcuma* cultivation is extensively found in tropical and subtropical regions of Asia, Australia, and South America (52). Since ancient times in India and China, it has been considered to treat illnesses such as dermatologic diseases, infection, stress, and depression (53–55).

The main part of the plant is the rhizomes, and the most prevalent active components are the curcuminoids (curcumin, demethoxycurcumin, and bisdemethoxycurcumin) (53, 56, 57). Curcuminoids are nontoxic polyphenolic that exerts a wide range of biological activities (58, 59), such as the production of significant immunosuppressants that inhibit the production of IL-2 and IL-12. This compound inhibits the expression of iNOS (inducible nitric oxide synthase), COX-2 (cyclooxygenase-2), lipoxygenase-5, and many other pro-inflammatory cytokines, such as TNF-α, IL-1, IL-6, and IL-8 (60–62).

Curcuminoids can also regulate apoptosis and suppress neurotoxic factors in macrophages and alveolar monocytes stimulated by lipopolysaccharides. Besides, it inhibits phosphorylation and degradation of IκBα (nuclear factor of kappa light polypeptide gene enhancer in B-cells inhibitor, alpha) and activates the γ receptor mechanism activated by peroxisome proliferator, reducing inflammation pattern induced by NF-κB pathway (62–64).

**Figure 3:**
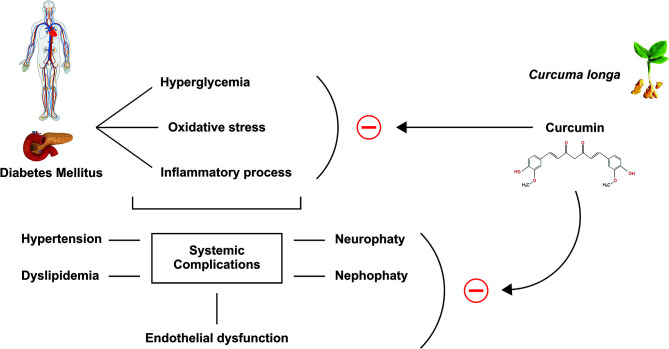
Effects of curcumin. Curcumin can inhibit hyperglycemia, oxidative stress, and the inflammatory processes caused by Diabetes Mellitus, in addition, and consequently inhibits the systemic complications of this disease, such as hypertension, dyslipidemia, neuropathy, nephropathy, and endothelial dysfunction.

The peculiar characteristics that attract scientists’ attention are the antioxidant and anti-inflammatory activities and the safety of its pharmacological profile (14, 15, 65, 66). The mechanism of action in several molecular pathways is due to curcumin’s particular chemical structure, capable of having many molecular targets. The biological effects may include the inhibition of reactive oxygen species (ROS) production, playing a fundamental role, particularly for diseases related to oxidative stress and inflammation, such as DM (67, 68). **Figure 3** shows some systemic effects of curcumin.

As seen above, curcumin can exhibit a huge range of therapeutic possibilities; however, it presents low solubility and rapid metabolism limiting its absorption in the gastrointestinal tract and resulting in weak bioavailability. The weak bioavailability has been associated to its insolubility in the water and the increased degradation in alkaline solutions or crystallization in acidic environments (69–71). Significant changes occur in its degradation time according to the pH, at pH> 7 if it degrades in about 30 min, already in acidic conditions, the degradation is much slower, with less than 20% of the total curcumin decomposed in 1 h. When ingested orally, the bioavailability of curcumin can be influenced by the food matrix, like lipids and proteins. Most of it is excreted in the feces, and only a small part will be absorbed in the intestine, which still undergoes a fast metabolism in the liver and the plasma. A high conjugation rate *via* glucuronidation and sulfation is also largely converted to its water-soluble metabolites (sulfates and glucuronides) that are excreted in the urine, explaining its concentration has a very low level in the blood (72–74).

The low bioavailability leads to reduced serum concentrations, reducing the possibilities of producing positive health effects. Due to these reasons, in the last few years, several delivery methods have been developed to improve oral curcumin bioavailability (75, 76). Some pharmaceutical technologies, and combinations with other compounds, such as piperine or lecithin, are being considered as they increase curcumin´s solubility, extend its residence in plasma, and improve the pharmacokinetic profile and cell absorption. Different new delivery systems, such as solid lipid particles, micellar systems, or hydrophilic nanoparticles, can increase curcumin concentration up to 15 to 20 times. Therefore, there is an improved curcumin solubility, bioavailability, transmembrane permeability, prolonged plasma half‐life, long‐term stability, target‐specific delivery, and upgraded therapeutic effects (77, 78).

According to studies that explored the safety of turmeric, standardized powder and extract of turmeric and curcumin are safe for human use, even in high doses of 6 g/day for seven weeks. Moreover, endovenous use of curcumin is safe if used if the dose administered is lower than that used orally. In pregnant animals, curcumin showed to be safe, but further studies are needed to confirm its safety in pregnant women (79, 80). Curcumin is also a nontoxic, non-mutagenic, non-carcinogenic, non-photo toxic agent and considered safe at lower doses than oral doses in intravenous administration in humans.

Nevertheless, some adverse effects are related to its consumption, such as dyspepsia, nausea, flatulence, and diarrhea. It was also demonstrated that turmeric could interact with some medications; it affects cytochromes P450, and the pharmacokinetics of some conventional drugs such as anticoagulants, antibiotics, antidiabetics, cardiovascular drugs, anticancer drugs, and antidepressants are influenced by curcuminoids (52, 80–82).

### Effects of *Curcuma longa* and Curcumin on Type 2 Diabetes Mellitus

Several studies have investigated the effects of curcumin on diabetes. These studies are shown in **Table 1** and discussed below.

A Pilot Study performed in Mexico (28) showed evidence that dietary supplementation with curcumin can reduce oxidative stress in patients with non-diabetic or diabetic proteinuric CKD. Curcumin significantly improved the elimination of free radicals activity in individuals with non-diabetic proteinuric CKD and reduced the plasma levels of malondialdehyde (MDA). However, its effects were limited; patients with diabetic proteinuric CKD treated with placebo showed a significant reduction in MDA content after the intervention. Also, no significant differences were observed in glutathione disulfide (GSSG) content or glutathione (GSH)/GSSG ratio between groups or with relation to sampling. Curcumin treatment did not modify the antioxidant activity of glutathione peroxidase, glutathione reductase, erythrocyte superoxide dismutase (SOD), and erythrocyte catalase, or to increase nuclear factor (erythroid-derived 2)-like 2 (Nrf2) activation in both patients with non-diabetic and diabetic proteinuric CKD. A study with a higher dose and longer follow-up is necessary to confirm these findings.

Another study, which investigated the effect of nano-curcumin in T2DM individuals, was adequately randomized, with no significant loss of participants, and even with a dose that the authors considered low, the effects of curcumin were positive. The fasting blood glucose (FBG), Hemoglobin A1c (HbA1C), body mass index (BMI), Estimated Average Glucose (eAG), total cholesterol (TC), LDL-c, HDL-c, and triglyceride (TG) were compared between the two groups after the intervention. Curcumin improved FBG, HbA1c, BMI, and eAG, but did not affect LDL-c, HDL-c, TG, and TC (29).

The results of Panahi et al. (20) showed an antioxidant effect of curcuminoid supplementation in patients with T2DM since supplementation promoted a significant increase in total antioxidant capacity and serum SOD activities, while MDA amounts were significantly decreased compared to the placebo group. Future studies may assess the impact of these antioxidant effects on diabetic complications and cardiovascular outcomes.

The treatment of T2DM patients with curcuminoid plus piperine resulted in a reduction in serum Lp (a) and an increase in HDL-c concentrations. These results are important because, until very recently, the possibilities of influencing Lp (a) were extremely limited. Serum concentrations of lipids including TC, LDL-c, HDL-c, TG, lipoprotein (a) (Lp(a)), and non-HDL-c were investigated at the beginning and at the end of the trial and revealed significant reductions in serum TC, non-HDL-c, and Lp (a) levels, and increase in serum HDL-c levels in the curcuminoid group compared to the placebo. Therefore, curcuminoids plus piperine may be a useful supplement in treating dyslipidemia in patients with T2DM (21).

The trial results performed by Panahi et al. (30) also revealed a beneficial effect of curcuminoids plus piperine supplementation on glycemic and hepatic parameters but not on hs-CRP levels in T2DM patients. However, this study has insufficient information regarding standard-of-care treatment that may be involved in the lack of curcuminoid efficacy on some parameters measured, particularly hs-CRP. Second, dietary intake and physical activity were not assessed; nevertheless, by randomization, it is expected that these characteristics were distributed similarly in the study groups minimizing the risk of bias. Finally, the trial duration was short, and it would help assess the efficacy of curcuminoids in long-term trials.

Positive effects of curcumin were also found by Adab et al. (31) in hyperlipidemic T2DM patients who received turmeric powder daily for eight weeks. BMI, TC, TG, LDL-c, HDL-c, insulin, HbA1C, FBG, fasting serum insulin, Apolipoprotein A1 and B were evaluated at the beginning and after the period of intervention. The authors concluded that this intervention could be used as an adjunct therapy to reduce diabetes complications, atherosclerosis, and overweight. The limitations of this trial may include a short intervention duration and low sample size.

The trial performed by Adibian et al. (32) with T2DM patients showed that the intervention with 1,500 mg daily of curcumin for ten weeks might reduce diabetes complications by decreasing indicators of inflammation. However, the authors suggest that higher sample sizes, longer duration, and different curcumin doses could lead to better outcomes.

In the trial performed by Asadi et al. (33), supplementation with nano-curcumin in patients with T2DM improved and reduced the severity of DSPN. The study also showed a reduction in glycemia after 2 h (Bs2hp), weight, and body mass index; however, these modifications were not significant. Meanwhile, the authors recognize some limitations: a short duration of follow-up, a single-dose trial, and low male participation.

The randomized, double-blind placebo-controlled trial performed in Iran by Hodaei et al. (34) indicated that the daily administration of a high dose of curcumin for ten weeks in NIDDM patients had positive effects on inflammatory indicators. However, this intervention had no effects on oxidative stress, serum insulin levels, IR, and HbA1c. The authors recognize that the study had a short duration of intervention and showed significant loss of patients.

Srinivasan et al. (35) showed that intervention with *Curcuma longa* produced a significant reduction in arterial stiffness in T2DM patients. The carotid-femoral pulse wave velocity (PWV), left brachial-ankle PWV, aortic augmentation pressure, aortic augmentation index, and aortic augmentation index decrease significantly. The authors attributed the lack of statistical reduction in endothelial dysfunction markers as adiponectin, leptin, ICAM, and VCAM due to methodological analysis limitations.

In the trial performed by Vanaie et al. (36), curcumin’s oral administration showed benefit on renal function by significantly decreasing albuminuria, therefore being considered a promising alternative therapy for T2DM patients with a mild decrease in glomerular filtration rate (GFR). The authors recognized some study limitations, such as small sample size and short intervention time.

The treatment of patients with T2DM with nano-curcumin capsules showed a beneficial effect on depression and anxiety. Moreover, curcumin was safe and well-tolerated during the study (37). However, these effects were minor, and no significant effect was observed for stress. The authors considered that the intervention might show better effects in the long term or higher supplementation doses.

Funamoto et al. (38) showed that the oral intervention with Theracurmin^®^ in patients with impaired glucose tolerance or non-insulin-dependent diabetes mellitus could increase adiponectin expression, resulting in antiatherosclerotic action. Besides, the authors noticed that leptin demonstrated a decreasing trend after administration of Theracurmin. However, this trial’s limitations were the small sample size and the short duration of the intervention.

The results of Shafabakhsh et al. (39) revealed that the intervention with nano-curcumin in T2DM significantly decreased fasting plasma glucose and insulin levels and decreased the plasma lipids. Also, nano-curcumin intake upregulated gene expression of PPAR-γ and LDLR in PBMCs and increased total nitrite and total antioxidant capacity (TAC) levels without affecting GSH levels and gene expression of TGF-β. The authors concluded that this supplementation showed anti-inflammatory and antioxidant effects; however, they recognized some limitations in the trial, since they did not check compliance to nano-curcumin intake, and they were also unable to determine the effects of the administration on other biomarkers of oxidative stress and inflammation.

Another clinical trial developed by Shafabakhsh et al. (40) showed that curcumin intake upregulated PPAR-gamma in peripheral blood mononuclear cells of T2DM subjects with coronary heart disease (CHD). Furthermore, it was able to improve Pittsburgh Sleep Quality Index (PSQI) score. The authors considered that this is the first analysis of curcumin’s effects on psychological symptoms, inflammatory factors, oxidative status, and gene expression related to metabolic profiles in patients with T2DM and CHD. These findings suggest that curcumin can improve the psychological status, metabolic disorders, and patients’ quality of life. The study’s limitations include the short period of intervention, small sample size, lack of examinations for other metabolic and genetic biomarkers of inflammation, and oxidative damage.

The clinical trial developed by Mokhtari et al. (41) showed that the oral intervention with nano-curcumin in patients with diabetic foot ulcer (DFU) resulted in a significant improvement of FPG, insulin levels, the homeostasis model of assessment-insulin resistance (HOMA-IR), the quantitative insulin sensitivity check index (QUICKI), LDL-c, TAC and total glutathione (GSH) levels, but did not affect ulcer size, HbA1c, lipid profile, markers of inflammation, and oxidative stress. Therefore, the study revealed strengths, such as the first study that evaluated the effects of nano-curcumin on wound healing parameters and metabolic control in DFU patients. As DFU patients are susceptible to insulin resistance and cardiometabolic disorders, there was a good rationale for this project. However, the trial had few limitations, including the small sample size and the short intervention period. Furthermore, the authors could not assess the effects of curcumin supplementation on gene expression related to insulin resistance, lipid homeostasis, and inflammation in patients with DFU.

The trials that met the eligibility criteria for this review showed that curcumin significantly improves insulin resistance, serum glucose levels, HbA1c, lipid profile, and inflammatory biomarkers in patients with T2DM. However, in low doses and a short period of use, it may not interfere with the disease’s symptoms. Despite this, as T2DM remains incurable, understanding the role of curcumin in this pathology may represent a new therapeutic target.

## Conclusion

T2DM has a multifactorial pathology and affects thousands of people worldwide. Its treatment consists of lifestyle changes, diet, physical activity, and therapies with medications for the rest of life. Curcumin is a natural anti-inflammatory and anti-diabetic agent representing a safe and low-cost alternative for this condition’s therapeutic approach, although it is still necessary to know its effective dose. We suggest that more robust and rigorous randomized controlled clinical trials are carried out to establish the role of curcumin in the therapeutics of T2DM.

## Data Availability Statement

The original contributions presented in the study are included in the article/supplementary material. Further inquiries can be directed to the corresponding author.

## Author Contributions

All the authors contributed equally to the manuscript. All authors contributed to the article and approved the submitted version.

## Conflict of Interest

The authors declare that the research was conducted in the absence of any commercial or financial relationships that could be construed as a potential conflict of interest.
